# Genomic and transcriptomic characterization of pre-operative chemotherapy response in patients with osteosarcoma

**DOI:** 10.1038/s41598-023-46857-8

**Published:** 2023-11-27

**Authors:** Yongkun Yang, Zhen Huang, Mingming Yuan, Jinqiu Rui, Rongrong Chen, Tao Jin, Yang Sun, Zhiping Deng, Huachao Shan, Xiaohui Niu, Weifeng Liu

**Affiliations:** 1 Department of Orthopaedic Oncology Surgery, Beijing Jishuitan Hospital, Capital Medical University, No. 31, Xinjiekou East Street, Xicheng District, Beijing, 100035 China; 2 Fourth Medical College of Peking University, Beijing, 100035 China; 3 Geneplus-Beijing, Beijing, 102206 China

**Keywords:** Bone cancer, Predictive markers, Translational research

## Abstract

Osteosarcoma is a heterogeneous disease with regard to its chemotherapy response and clinical outcomes. This study aims to investigate the genomic and transcriptomic characteristics related to pre-operative chemotherapy response. Samples from 25 osteosarcoma patients were collected to perform both whole exome and transcriptome sequencing. Osteosarcoma had significant amount of chromosomal copy number variants (CNVs). Chemotherapy responders showed the higher chromosomal CNV burden than non-responders (p = 0.0775), but the difference was not significant. The percentage of COSMIC signature 3, associated with homologous recombination repair deficiency, was higher in responders (56%) than in non-responders (45%). Transcriptomic analysis suggested that 11 genes were significantly up-regulated in responders and 18 genes were up-regulated in non-responders. Both GSEA and KEGG enrichment analysis indicted that four pathways related to cardiomyopathy were up-regulated in responders, while neuroactive ligand − receptor interaction was up-regulated in non-responders. Finally, a previously published chemoresistant model was validated using our dataset, with the area under the curve of 0.796 (95% CI, 0.583–1.000). Osteosarcoma had the heterogeneous mutational profile with frequent occurrence of CNVs. Transcriptomic analysis identified several signaling pathways associated with chemotherapy responsiveness to osteosarcoma. Transcriptomic signatures provides a potential research direction for predicting the chemotherapy response.

## Introduction

Osteosarcoma is the most common primary malignant tumor of bone. Although it is a rare disease in all ages, primary osteosarcoma commonly occurs in children and adolescents aged 10–24 years. According to the statistical analyses of 5 016 osteosarcoma patients from the Surveillance, Epidemiology, and End Results program from 1975 to 2017^1^, the age-adjusted incidence of osteosarcoma was 3.3/1,000,000 in all age groups and 7.2/1,000,000 in population aged 10–24 years. Pre-operative (neoadjuvant) chemotherapy followed by definite surgery and postoperative (adjuvant) chemotherapy is the mainstay of therapy for osteosarcoma, significantly improving the survival and prognosis in approximately two-thirds of patients with localized disease^2^.

Despite the remarkable efficacy of chemotherapy, there is a lack of uniform and objective criteria for evaluating the chemotherapy response in osteosarcoma patients. How to evaluate the effectiveness of chemotherapy, especially pre-operative chemotherapy, is crucial for the adjustment of treatment plans and patient prognosis assessment. Currently, the commonly applied evaluation method to determine the chemotherapy response and tumor prognosis is based on the tumor cell necrosis rate^3,4^. However, the evaluation and application of tumor cell necrosis rate have certain limitations: the procedure of tumor necrosis rate analysis is complex; heterogeneous tumors are difficult to carry out; the evaluation time required is long, resulting in a late outcome which is not conducive to subsequent treatment. Huvos^5^ first proposed a histological evaluation method for osteosarcoma necrosis rate in the 1970s, which has been used for more than 40 years. However, this method cannot be applied to evaluate the chemotherapy response before surgery, and it does not comprehensively consider clinical and imaging aspects. Therefore, it is necessary to understand the molecular characteristics related to chemotherapy response and find a new effective evaluation method for chemotherapy response in osteosarcoma.

The rapid development and widespread application of high throughput sequencing techniques promote the understanding of tumor development and progression from the molecular level, and thus change the clinical treatment modes and survival outcomes of patients in various of cancers, especially in non-small cell lung cancer and colorectal cancer^6,7^. In addition to supporting treatment decisions for cancer patients, next-generation sequencing has also been utilized in multiple clinical settings, including detecting tumor susceptibility genes, early diagnosis and screening, evaluating patient prognosis and monitoring minimal residual diseases and tumor progression^8–11^. Zeng et al.^12^ developed a chemoresistant risk model using markers ontained from bulk RNA and single-cell RNA sequencing with the area under the curve of 0.82 in the TARGET-OS training cohort and 0.84 in the GSE33382 validation cohort. This study aims to investigate the genomic and transcriptomic characteristics of osteosarcoma using DNA- and RNA-based next-generation sequencing techniques, find clinical and molecular factors related to chemotherapy response and validate the performance of the previous model to predict chemotherapy response.

## Results

### Clinicopathologic characteristics

The clinicopathologic features of 25 patients with pathologic diagnoses of osteosarcoma were summarized in Supplementary Table S1. The median age at diagnosis was 15 years (range 8–32 years) and 75% patients were male. The most common site of osteosarcoma in this study was femur (60%), followed by tibia (20%), humerus (12%), fibula (4%) and multifocal (4%). Majority of the patients were conventional osteosarcoma (84%) and Surgical Staging System (SSS) stage IIB (92%). The tumor volume ranged from 24.633 to 14,705.856 cm^3^ with the median of 371.57 cm^3^. Pre-operative chemotherapy and surgery were conducted in 84% and 96% of patients, respectively. Pathological evaluation suggested that the tumor necrosis rate was higher than 90% in 40% of patients.

### Genomic landscape of osteosarcoma

**Figure 1:**
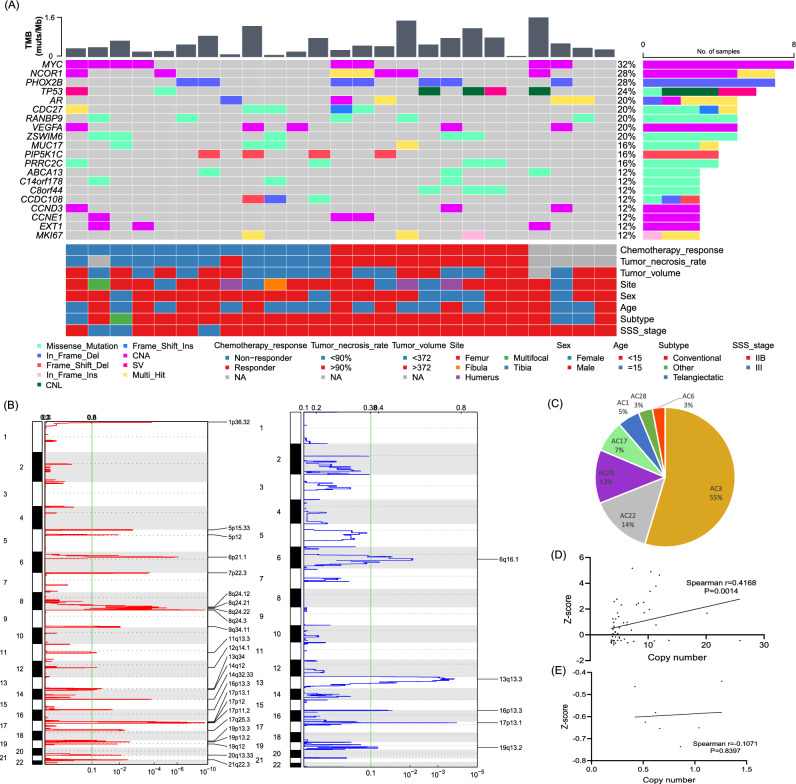
The genomic characteristics of osteosarcoma. (**A**) Clinical information and mutational landscape of 25 patients with osteosarcoma; (**B**) recurrent focal amplifications (left panel) and deletions (right panel) with the G-score and q values on the above and bottom of the figures, respectively; (**C**) inference of the Catalogue of Somatic Mutations in Cancer (COSMIC) mutational signatures composition in osteosarcoma; (**D**) the correlation between copy number of copy number amplifications on the genomic level and corresponding RNA expression on the transcriptomic level; (**E**) the correlation between copy number of copy number deletions on the genomic level and corresponding RNA expression on the transcriptomic level. CNA, copy number amplification; CNL, copy number loss; HRR, homologous recombination repair; MMR, mismatch repair; NA, not available; SSS, Surgical Staging System; SV, structural variant; TMB, tumor mutation burden.

A total of 1570 somatic mutations were detected in 25 patients with osteosarcoma, including 1488 single nucleotide variants and small insertions and deletions, 72 CNVs and 7 structural variants. The median number of mutations was 49 (range 2–215). *MYC* (8q24.21), *NCOR1* (7p12-11.2), *PHOX2B* (4p13) and *TP53* (17p13.1) were the top 4 commonly mutated genes, and mutated in 32%, 28%, 28% and 24% of osteosarcomas, respectively (Fig. 1A). Osteosarcoma had the extremely low level of TMB (median 0.47 muts/Mb, range 0.04–1.62 muts/Mb) compared with other tumors^13^, and significant amount of chromosomal CNVs (median: 5, range 0–15). A total of 25 significantly amplified and 5 deleted regions were identified with the q value < 0.1 (Fig. 1B). COSMIC mutational signature analysis showed that osteosarcoma was mainly associated with signature 3 (55%, failure of DNA double-strand break-repair by homologous recombination), 22 (14%, aristolochic acid exposure) and 20 (13%, defective DNA mismatch repair) (Fig. 1C). To examine the relationship between copy number and expression level of CNVs, Spearman correlation analysis was performed and the result indicated that copy number for amplifications was positively correlated with Z-score, with the Spearman coefficient of 0.4168 (p = 0.0014) (Fig. 1D). The Spearman coefficient for deletions was -0.1071 (p = 0.8397) for (Fig. 1E), respectively.

**Figure 2:**
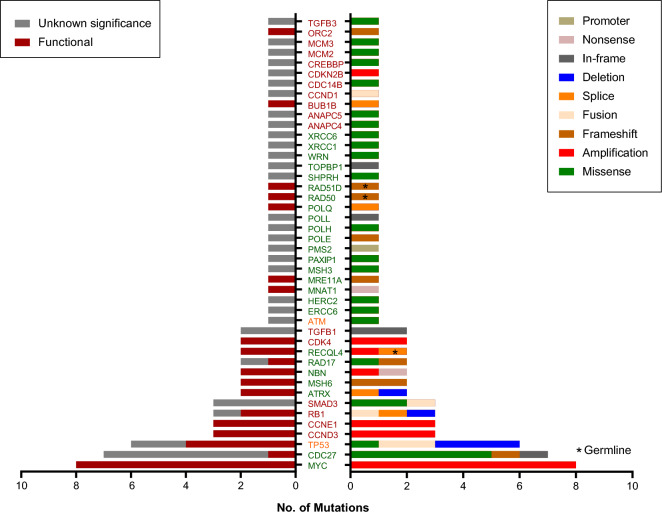
Mutations in DNA damage repair and cell cycle pathways. Genes belonging to DNA damage repair, cell cycle, and both of them were represented using green, red and orange color, respectively.

Due to the association between signature 3 and 20 and osteosarcoma, we further analyzed the mutations on genes involved in DNA damage repair (DDR) and cell cycle pathways (Fig. 2). DDR and cell cycle pathway mutations were detected in 52% and 84% of osteosarcoma patients, and the functional mutations (including activating missense mutations, truncated mutations, amplifications and deletions) were detected in 40% and 56% of patients, respectively. The common functional mutations in these two pathways included *MYC*, *CCND3*, *CCND1* amplifications and *TP53* deletions.

### Relationship between genomic features and chemotherapy response

**Figure 3:**
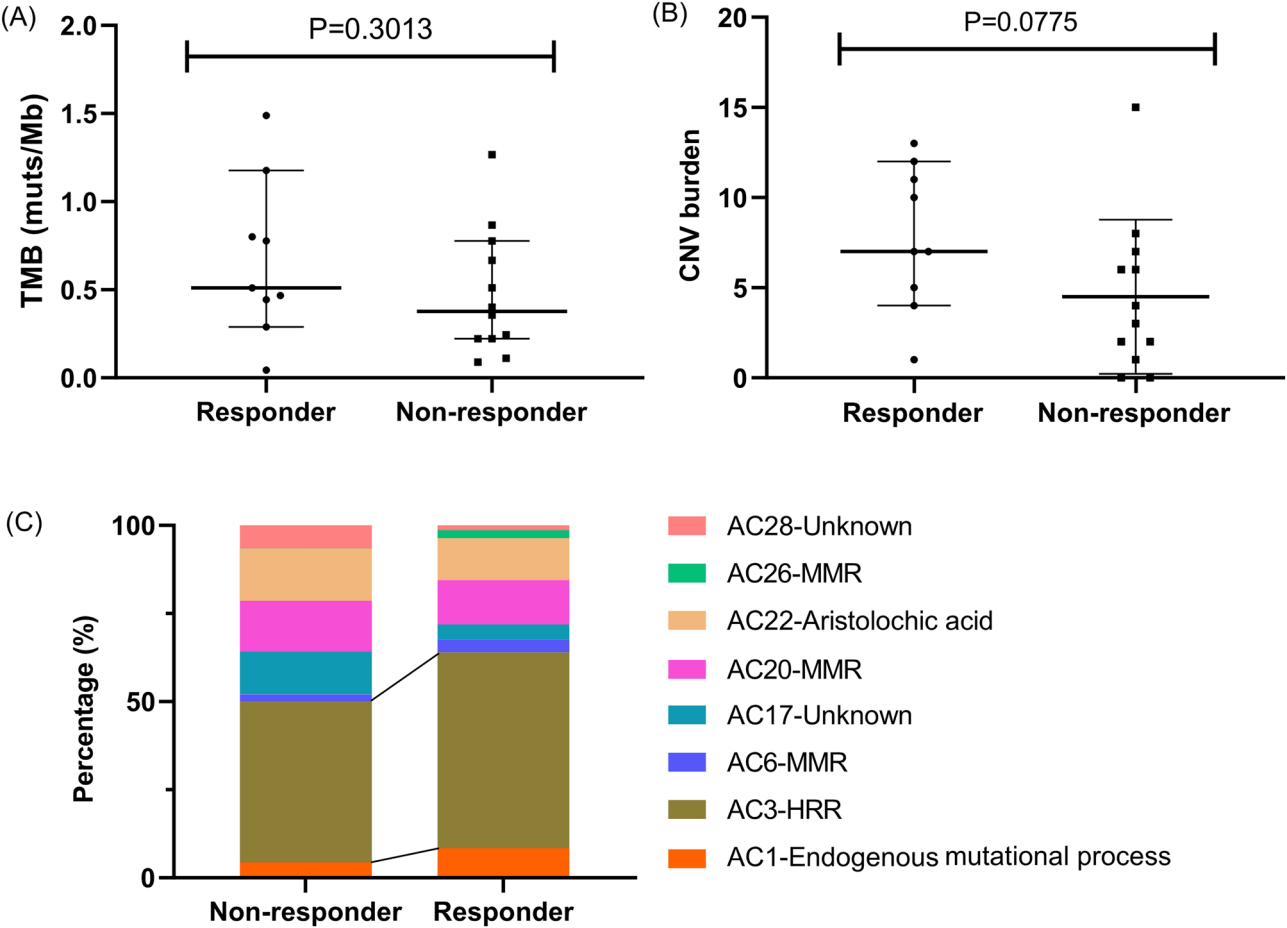
The relationship between genomic features and chemotherapy response. (**A**) Comparison of TMB between chemotherapy responders and non-responders; (**B**) comparison of CNV burden between chemotherapy responders and non-responders; (**C**) comparison of the Catalogue of Somatic Mutations in Cancer (COSMIC) mutational signatures composition between chemotherapy responders and non-responders. CNV, copy number variant; HRR, homologous recombination repair; MMR, mismatch repair; TMB, tumor mutation burden.

To explore the relationship between genomic characteristics and chemotherapy response, fisher’s exact test was used to find the differentially mutated genes between responders and non-responders with significance, and the result was negative, which may be explained by the low mutation rate in osteosarcoma (data not shown). Additionally, no difference in TMB between two groups was observed (median TMB for responders vs. non-responders: 0.5111 vs. 0.3778 muts/Mb, p = 0.3013) (Fig. 3A), and the relationship between TMB and survival time was not investigated in this study. Notably, the chromosomal CNV burden of chemotherapy responders appeared to be higher than that of non-responders with the median of 7 and 3.5, respectively, but the difference was not statistically significant (p = 0.0775) (Fig. 3B). The composition of COSMIC mutational signatures was also compared between chemotherapy responders and non-responders using chi-square test and the p value was smaller than 0.0001 (Fig. 3C). Mutational signatures 1, 3, 6, 17, 20, 22 and 28 were present in both groups. Apparently, the percentage of signature 3, which was associated with failure of DNA double-strand break-repair by homologous recombination, was higher in chemotherapy responders (56%) than in non-responders (45%), which suggested that signature 3 may be related to chemotherapy response. By contrast, the percentage of two signatures of unknown etiology (signature 17 and 28) was higher in non-responders (Non-responders vs. Responders: 12% vs. 4% for signature 17; 6% vs. 1% for signature 28).

### Differentially expressed genes (DEGs) and pathway enrichment analysis

**Figure 4:**
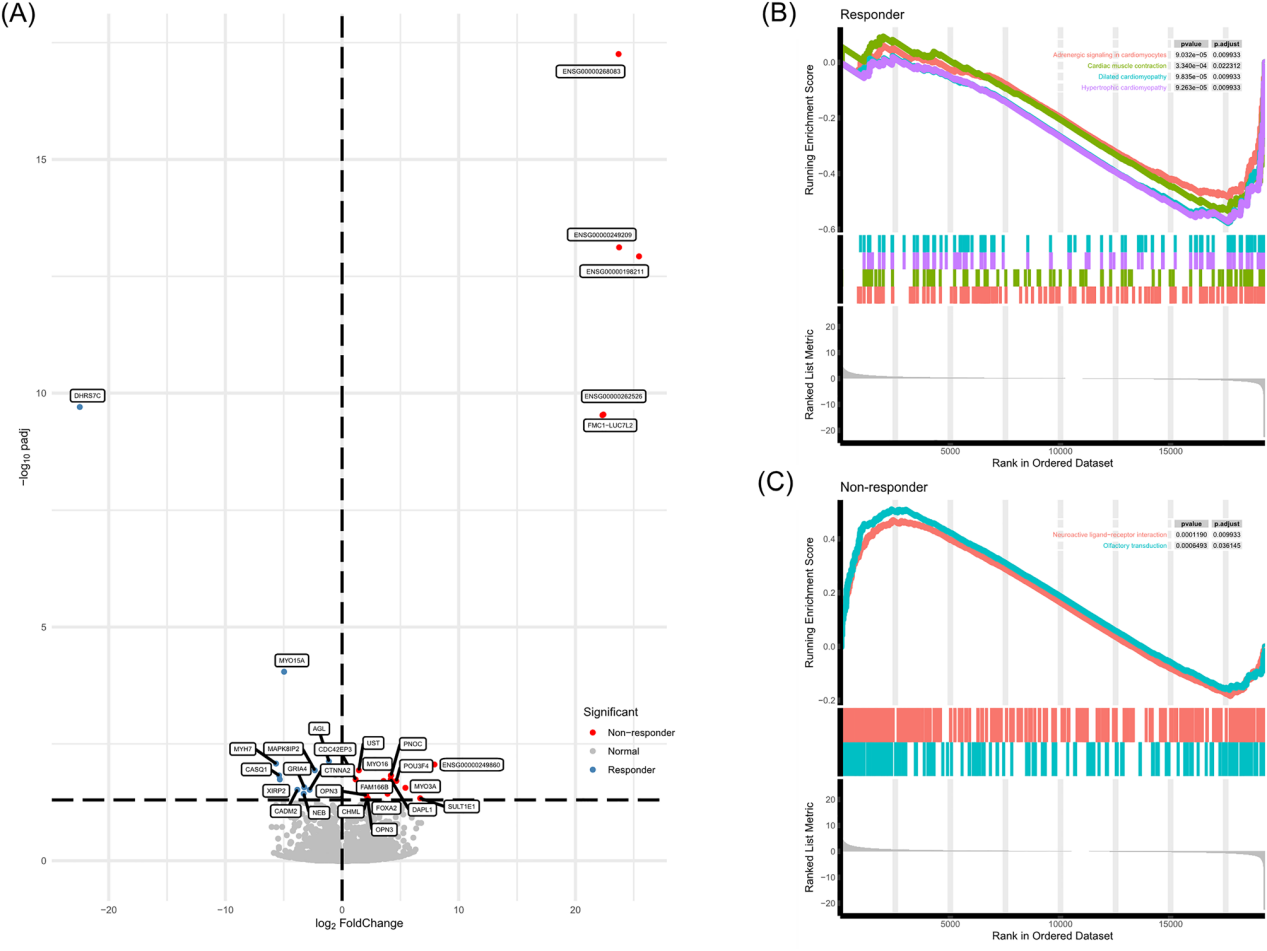
Differentially expressed genes and enrichment analysis between chemotherapy responders and non-responders. (**A**) Volcano plot showing the differentially expressed genes between groups; GSEA analyses of genesets for responders (**B**) and non-responders (**C**).

The raw expression counts for all genes were executed for the differentially expressed gene analysis between chemotherapy responders and non-responders. The thresholds for adjusted p (padj) and |log2FC| were set as < 0.05 and ≥ 1, respectively. As a result, the volcano plot showed that 11 genes were significantly up-regulated in responders and 18 genes were significantly up-regulated in non-responders with the adjusted p value smaller than 0.05 (Fig. 4A). GSEA enrichment analysis was performed to identify the up-regulated pathways in chemotherapy responders and non-responders. Four pathways related to cardiomyopathy were up-regulated in responders (Fig. 4B), while neuroactive ligand − receptor interaction and olfactory transduction pathways were up-regulated in non-responders (Fig. 4C).

**Figure 5:**
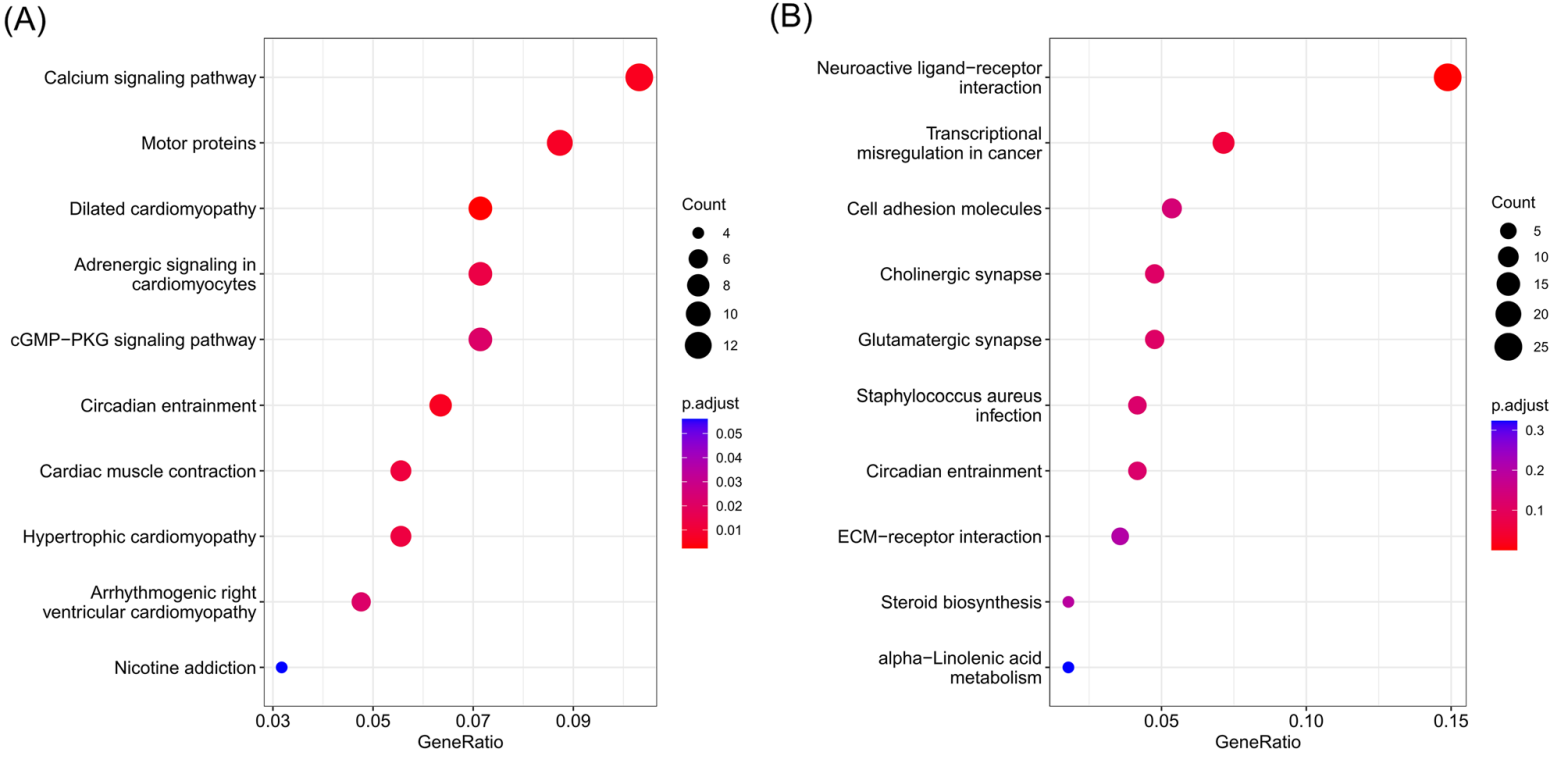
Dotplots showing the KEGG pathway enrichment analysis of genes differentially expressed in (**A**) chemotherapy responders and (**B**) non-responders.

Due to the relative small number of DEGs, genes with p < 0.05 and |log2FC|≥ 1 were included to perform conventional KEGG pathway enrichment analysis^14–16^ and PPI analysis. The significant KEGG pathways (adjusted p < 0.05) of 309 significantly expressed genes in responders included calcium signaling pathway, motor proteins, cGMP − PKG signaling pathway, circadian entrainment, arrhythmogenic right ventricular cardiomyopathy, as well as 4 pathways identified by GSEA enrichment analysis (Fig. 5A). In non-responders, neuroactive ligand-receptor interaction pathway was the only significant KEGG pathway with the adjusted p value smaller than 0.05 (Fig. 5B). Genes involved in these pathways were summarized in Supplementary Table S2. The PPI analysis was performed using STRING database and visualized by Cytoscape (Supplementary Fig. 1). A total of 709 nodes and 1888 edges were included in the network. *ACTN2*, *TTN* and *MYH6* were considered as potential hub genes with the node degree score of 45, 43 and 39, respectively. *ACTN2*, *TTN* and *MYH6* were overexpressed in chemotherapy responders with the |log2FC| of 1.8487, 3.6915, 2.1881 and p value of 0.0103, 0.0001, 0.0200, respectively (data not shown). In addition, all of them were involved in pathways enriched in chemotherapy responders (Supplementary Table S2).

### Model validation to predict chemotherapy response

**Figure 6:**
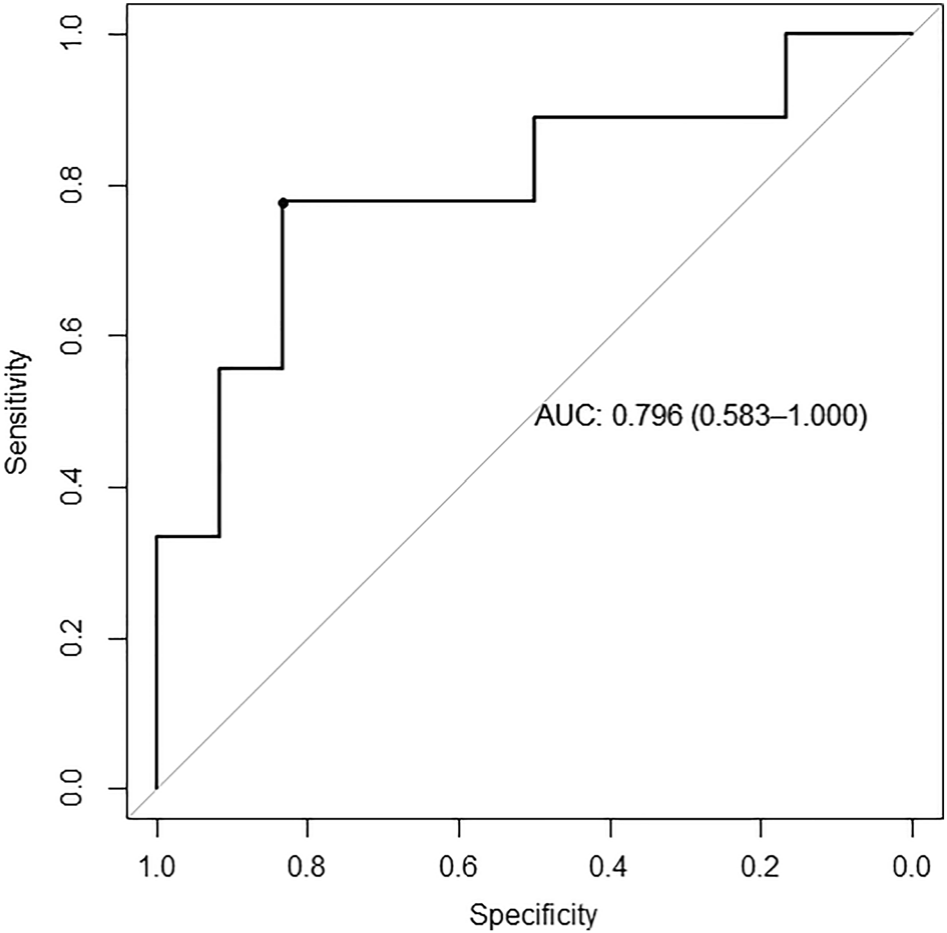
Receiver operator characteristic curve of the published model to predict chemotherapy response using our dataset.

Zeng et al.^12^ developed a robust chemoresistant risk model using the expression levels of 10 genes. The performance of this model to discriminate chemotherapy response was validated using our data. The chemoresistant risk score was calculated using the expression level of 10 genes according to the formula: Risk score =  − 36.36 + 0.110 ∗ ADAMTS2 + 0.042 ∗ SPAG16 + 0.124 ∗ CGREF1 + 0.328 ∗ JTB + 0.083 ∗ ENPP2 + 0.528 ∗ ACP1 + 1.485 ∗ NPM1 + 0.759 ∗ CTSF + 0.045 ∗ MPP6 + 1.109 ∗ PARD6G. The model had the area under the curve of 0.796 (95% confidence interval, 0.583–1.000) (Fig. 6), suggesting the acceptable performance.

## Discussion

Osteosarcoma is a heterogeneous disease with regard to its histology, chemotherapy response and clinical outcomes^17,18^. In this study, whole exome and transcriptome sequencing were conducted in 25 patients with osteosarcoma. Genomic and transcriptomic features relevant to chemotherapy response were explored and a previous chemoresistant model was validated. To our knowledge, this is the first study to comprehensively investigate the clinical and molecular characteristics of chemotherapy response in osteosarcoma using multi-omics techniques, which expands our knowledge of this complex disease.

In multiple previous studies^19–21^, osteosarcoma was mainly characterized by diverse variants, recurrent structural variants, high frequencies of *TP53* and *RB1* mutations. Similarly, our study revealed that the genetic variants in osteosarcoma were dispersedly distributed. In terms of mutation type, single nucleotide variants and small insertions and deletions were atypical events, whereas CNVs, especially amplification, were common in osteosarcoma. *PHOX2B* mutations were firstly identified in osteosarcoma in our study, which encodes a transcription factor participating in the development of the peripheral nervous system and is known to be related to neuroblastoma and congenital central hypoventilation syndrome^22^. In our study, all *PHOX2B* mutations were in-frame deletions occurred in the 20 polyalanine region of *PHOX2B* (i.e. polyAla contractions) and evaluated as benign/likely benign variants by ClinVar (Variation ID: 227016). Therefore, they are more likely to be passenger mutations. Our study also found that osteosarcoma had the low level of TMB, which was consistent with previous studies showing the median TMB ranged from 0.1 to 2.5 muts/Mb^13,23–27^. In addition, osteosarcoma had significant amount of chromosomal CNV. It seemed that patients with high chromosomal CNV burden had a nonsignificantly better response to chemotherapy, which required to be verified in further studies. Consistent with the previous study^20^, mutations in osteosarcoma were mainly caused by the dysfunctional DNA damage repair in this study. Moreover, this study suggested that chemotherapy responders had more homologous recombination repair deficiency—induced mutations, which may be explained by the administration of platinum-based chemotherapy regimens in osteosarcoma and the favorable response to platinum in defective homologous recombination repair ovarian cancer^28^.

In this study, 11 genes were significantly up-regulated in responders and 18 genes were significantly up-regulated in non-responders. Both KEGG and GSEA enrichment analyses suggested that 4 pathways related to cardiomyopathy were significantly enriched in responders and neuroactive ligand-receptor interaction pathway was significant in non-responders. So far, no direct evidence has been found to support the relationship between cardiomyopathy and chemotherapy response. In a previous study, transcriptomic analysis was performed in breast cancer to investigate biomarkers related to chemotherapy sensitivity. KEGG analysis indicated that a great number of DEGs were enriched in neuroactive ligand-receptor interaction pathway. In addition, the number of DEGs in this pathway were more likely to be subexpressed in chemotherapy resistant group. However, whether the entire pathway was up-regulated or down-regulated was not analyzed^29^. In another study in breast cancer, the relationship between neuroactive ligand-receptor interaction pathway and pathological complete response to chemotherapy was not clearly elucidated^30^. One study used the paclitaxel-induced peripheral neuropathy rat model to understand the transcriptomic level of the dorsal root ganglia neurons and found that neuroactive ligand-receptor interaction was majorly involved in sensory neurons of rats with paclitaxel-induced peripheral neuropathy^31^. PPI analysis identified *ACTN2*, *TTN* and *MYH6* as hub genes resulting in chemotherapy responsiveness. *ACTN2* encoded protein links the anti-parallel actin filaments, contributes to sarcomere stability and is related to cardiomyopathy^32^. *TTN* encoded protein plays an important role in skeletal and in heart muscles^33^. *MYH6* gene provides instructions for making a protein known as the cardiac alpha (α)-myosin heavy chain, which forms part of type II myosin in cardiac muscles^34^. According to The Human Protein Atlas database, ACTN2, TTN and MYH6 are highly expressed in muscle cells. Their higher expression in the chemotherapy responders may indicate that the specimen was derived from an extra-osseous lesion that had invaded muscle, and tumors invading the surrounding muscle may be more sensitive to chemotherapy. However, this hypothesis can not be verified using histological or immunohistochemistry analysis as no sufficient tissue can be obtained, which requires further investigations.

In clinics, multiple methods are used to evaluate the neoadjuvant chemotherapy response in osteosarcoma, including symptoms and signs, laboratory tests, imaging examinations and evaluation of tumor necrosis rate. Zeng et al.^12^ developed a chemoresistant risk model with the area under the curve of 0.82 in the TARGET-OS training cohort and 0.84 in the GSE33382 validation cohort. Our study revealed an acceptable performance of this model with the AUC of 0.796. A potential limitation of this study was the small amount number of participants, which may reduce the representativeness of certain findings and need to be further verified in large-scale studies.

In conclusion, multi-omics sequencing techniques help us better understand the molecular characteristics of osteosarcoma. Osteosarcoma is a highly heterogeneous disease with frequent occurrence of CNVs at the genomic level. Transcriptomic analysis identified several signaling pathways associated with chemotherapy responsiveness to osteosarcoma, including pathways related to cardiomyopathy and neuroactive ligand-receptor interaction. Additionally, a model based on transcriptomic characteristics of osteosarcoma may be used for predicting the chemotherapy response in clinics..

## Materials and methods

### Patients and samples

A total of 25 patients with the definite diagnosis of osteosarcoma in Beijing Jishuitan Hospital (Beijing, China) from December 2021 to November 2022 were prospectively enrolled in this study. Clinical information including demographics, pathologic diagnoses, treatment history and imaging examinations were collected. Tumor tissue samples (including 6 fresh tissues, 18 formalin immersed tissues and 1 formalin fixed paraffin-embedded tissue) and 2 ml matched peripheral blood were collected from all participants to perform the whole exome and transcriptome sequencing. All procedures were conducted in accordance with the Declaration of Helsinki. This study was approved by the Ethics Committee of Beijing Jishuitan Hospital (Approval No. K2023013-00) and written informed consent was obtained from all participants.

### Evaluation of chemotherapy response

The evaluation of chemotherapy response mainly depended on tumor necrosis rate. The tumor cell necrosis rate was analyzed using the tumor tissues collected from tumor resection and separated into two groups: greater than 90% and less than 90%. As the lesion was suspected to be benign before surgery, one patient (P21) underwent surgery directly without preoperative chemotherapy. Three patients (P12, P15, P18) underwent unplanned surgery for other reasons and did not receive preoperative chemotherapy. Among 21 patients treated with chemotherapy, patients with tumor necrosis rates greater than 90% were considered as chemotherapy responders (N = 9), and those with tumor necrosis rates smaller than 90% were non-responders (N = 10). However, there were a few exceptions for the remaining two patients. P24 was initially diagnosed as AJCC stage IV osteosarcoma with lung metastases and not suitable for surgical resection. The tumor necrosis rate could not be assessed as the post-treatment tumor tissues were not available. Considering of the increase of the circumference difference of the tumor site between affected limb and healthy limb after chemotherapy, he was considered as a chemotherapy non-responder. For P14, the tumor necrosis rate assessed using small pieces of tumor tissues was greater than 90%. However, the clinical and imaging findings (including post-treatment increase of the circumference difference by 4.5 cm, unclear margin, increase of ossification and blood supply) suggested that the patient did not respond to chemotherapy. Therefore, the patient was finally evaluated as a non-responder by investigators. Taken together, 9 patients responded to chemotherapy, while 12 patients were non-responders.

### DNA- and RNA-based next-generation sequencing

Before sequencing, all tumor samples were treated with formalin to ascertain the tumor cell content. Whole exome and transcriptome sequencing were performed in Geneplus-Beijing (Beijing, China) as previously described^27–29^. Briefly, genomic DNA and RNA were extracted from the tumor samples using FirePureTM FFPE gDNA Extraction Kit for genomic DNA and RNeasy FFPE Kit for RNA or AllPrep DNA/RNA FFPE Kit for both DNA and RNA (QIAGEN, Hilden, Germany). Genomic DNA from leukocytes was extracted using the CWE9600 Blood DNA Kit (Cwbiotech, Taizhou, China). Sequencing libraries of genomic DNA and mRNA were prepared using KAPA DNA Library Preparation Kit (Kapa Biosystems, Wilmington, MA, USA) and NEB Next Ultra™ RNA Library Prep Kit (Illumina, Inc., San Diego, CA, USA), respectively. The DNA and RNA sequencing were performed using the DNBSEQ-T7RS High-throughput Sequencing platform (MGI, Shenzhen, China), whose performance was comparable to Illumina platform^35,36^. All experimental procedures followed the manufacturer’s instructions. Whole transcriptome sequencing cannot be performed for one patient due to the severe degradation of RNA. The detailed quality control data of DNA and RNA sequencing was provided in Supplementary Table S1.

### Bioinformatics analysis

After removal of terminal adaptor sequences and low-quality reads, the clean sequencing reads were aligned to the reference human genome (hg19) using BWA (version 0.7.10) and HISAT (version 2.0.4) for DNA and RNA sequencing, respectively. Genomic single nucleotide variants, small insertions and deletions, copy number variants (CNVs) and structural variants were detected using MuTect (version 1.1.4)/NChot, GATK (version 3.4–46-gbc02625) and CONTRA (version 2.0.8), respectively. Transcript assembly was performed using StringTie (version 1.2.3).

Tumor mutation burden was evaluated as the number of non-synonymous variants with the mutant allele frequency greater than 5% per megabase in the coding region. Chromosomal CNV burden represented the total level of amplifications or deletions at the chromosome level, which was calculated as previously described^30^. Gistic 2.0 was used to detect the significantly recurrent regions with amplification or deletion. The mutational landscape was portrayed using R package ‘maftools’ (version 2.14.0). To infer the composition of known Catalogue Of Somatic Mutations In Cancer (COSMIC) mutational signatures in osteosarcoma, R package ‘yapsa’ (version 1.24.0) was performed using the COSMIC mutational signatures version 2 (https://cancer.sanger.ac.uk/signatures/signatures_v2). Differentially expressed genes were analyzed using R package ‘DESeq2’ (version 1.38.3). R package ‘clusterProfiler’ (version 4.7.1.3) was used to perform gene set enrichment analysis (GSEA) enrichment analysis and conventional Kyoto Encyclopedia of Genes and Genomes (KEGG) pathway enrichment analysis. An online tool (STRING, https://string-db.org/) was used to analyze protein–protein interaction (PPI). The default confidence score > 0.40 was used to screen the PPI pairs. Cytoscape (version 3.9.1) was applied to visualize the PPI network. R package ‘pROC’ (version 1.18.0) was used to plot the receiver operator characteristic curve and calculate the area under the curve.

### Statistical analysis

Z-score normalization of gene expression data was performed using the scale function in R package ‘base’ (version 4.2.2) taking the raw count matrix as an input. Spearman correlation analysis was conducted to study the correlation between copy number and Z-score of CNVs. Differences of variables between groups were assessed using Mann–Whitney test for continuous variables and Fisher’s exact test or Chi-square test for categorical variables, with P < 0.05 considered as statistically significant.

### Ethics statement

All procedures were conducted in accordance with the Declaration of Helsinki**.** All the procedures involving human subjects were conducted following the ethical guidelines approved by the ethical committee of Beijing Jishuitan Hospital (Approval No. K2023013-00). All the participants have signed the informed consent for sequencing.

### Supplementary Information

Supplementary Information.

## Data Availability

The datasets generated and/or analysed during the current study are available in the Genome Sequence Archive (GSA) repository, [ACCESSION NUMBER: HRA005488].
