# Supplementary material for: Genomic and transcriptomic characterization of pre-operative chemotherapy response in patients with osteosarcoma
Source: Sci Rep. 2023 Nov 27;13:20914. doi: 10.1038/s41598-023-46857-8 (PMC10684554; doi:10.1038/s41598-023-46857-8)

Supplementary Table S1. Clinicopathologic characteristics of 25 patients with osteosarcoma

Characteristics	Patients (n=25)
Age at diagnosis-years	
Median (range)	15 (8-32)
Gender-No.(%)	
Male	19 (76%)
Female	6 (24%)
Tumor location-No.(%)	
Femur	15 (60%)
Tibia	5 (20%)
Humerus	3 (12%)
Fibula	1 (4%)
Multifocal	1 (4%)
Histological subtype-No.(%)	
Conventional	21 (84%)
Telangiectatic	3 (12%)
Osteosarcoma arising from giant cell tumor of bone	1 (4%)
SSS stage-No.(%)	
IIB	22 (88%)
III	3 (12%)
Tumor volume-cm^3	
Median (range)	371.58 (24.633-14705.856)
Chemotherapy-No.(%)	
Yes	21 (84%)
No	4 (16%)
Surgery-No.(%)	
Yes	24 (96%)
No	1 (4%)
Tumor necrosis rate-No.(%)	
<90%	10 (40%)

>90%	10 (40%)
NA	5 (20%)

Abbreviations: NA, not available

Supplementary Table S2. KEGG pathway enrichment analysis of differentially expressed genes between chemotherapy responders and non-responders

Supplementary Table S2. KEGG pathway enrichment analysis of differentially expressed genes between chemotherapy responders and non-responders

Group	ID	Description	GeneRatio	BgRatio	pvalue	p.adjust	qvalue	geneID
Responder	hsa05414	Dilated cardiomyopathy	9/126	96/8378	<0.0001	0.0024	0.0023	PLN/TPM2/MYH6/LAMA2/ATP2A1/DES/ADCY1/TTN/MYH7
Responder	hsa04814	Motor proteins	11/126	193/8378	0.0001	0.0070	0.0066	TPM2/MYH6/DNAH2/TNNI1/DNAI4/ACTA1/TNNI2/MYH2/TNNC2/MYO15A/MYH7
Responder	hsa04260	Cardiac muscle contraction	7/126	87/8378	0.0003	0.0121	0.0114	TPM2/MYH6/ATP2A1/TRDN/UQCRFS1/MYH7/ATP1A2
Responder	hsa05410	Hypertrophic cardiomyopathy	7/126	90/8378	0.0004	0.0124	0.0117	TPM2/MYH6/LAMA2/ATP2A1/DES/TTN/MYH7
Responder	hsa04261	Adrenergic signaling in cardiomyocytes	9/126	154/8378	0.0005	0.0136	0.0128	PLN/TPM2/MYH6/ATP2A1/ADCY1/AGTR1/PPP1R1A/MYH7/ATP1A2
Responder	hsa04022	cGMP-PKG signaling pathway	9/126	167/8378	0.0009	0.0214	0.0201	PLN/MYH6/ATP2A1/ADCY1/AGTR1/IRS4/MYLK2/MYH7/ATP1A2

Supplementary Table S3. The detailed quality control data of DNA and RNA sequencing

Supplementary Table S3. The detailed quality control data of DNA and RNA sequencing

Patient ID	Tutor cell content (%)	DNA sequencing							RNA sequencing									
		DNA concentration (ng/μl)	DNA total amount (ng)	Library total amount (ng)	Average sequencing depth on target without duplicated reads	Mapping rate	Fraction of target covered with >= 0.2 mean depth	Contamination Ratio	RNA concentration (ng/μl)	RNA total amount (ng)	RNA quality （DV200）	Mapping Rate(%)	rRNA Rate(%)	Exonic Rate(%)	Q30(%)	Q20(%)	Clean Reads	Clean Bases
P01	30	12.9	774	774	545	97.94%	98.82%	0.19%	110	1980	0.58	92.93	8.77	53.68	89.31	96.89	311586408	27923806282
P02	20	42.4	2544	800.088	568	97.80%	91.19%	0.46%	21	378	0.29	91.05	1.57	56.06	94.69	98.38	1156077914	1.03688E+11
P03	20	39.6	2376	799.92	607	96.62%	88.65%	0.08%	7.78	140.04	0.36	79.26	0.75	66.44	94.23	98.21	279730256	25113637849
P04	15	5.3	318	318	766	99.82%	98.18%	0.08%	6.12	110.16	0.691	90.34	0.58	50.65	96.08	98.9	425309426	38139383003
P05	10	48.85	4885	400.0815	768	99.92%	98.51%	0.76%	46.4	835.2	0.7	89.01	30.76	56.61	92.5	97.07	268278630	23926870971
P06	40	31	1860	800.11	588	97.32%	94.58%	0.21%	30.4	547.2	0.672	95.79	8.02	81.01	94.78	98.44	234391310	21050847875
P07	30	22.6	1356	400.02	783	99.80%	99.01%	0.09%	74.6	1342.8	0.851	92.68	0.77	51.65	95.32	98.7	307064418	27557550078
P08	80	56	6720	800.24	531	97.91%	85.00%	0.11%	61.4	1105.2	0.993	90.94	0.88	56.67	93.97	98.08	514895448	46234208642
P09	15	5.12	307.2	307.2	568	99.63%	98.88%	1.25%	58.6	1054.8	0.882	96.88	0.36	56.18	94.36	98.41	315229994	28321532886
P10	30	69.6	4176	799.704	741	99.20%	98.84%	0.13%	102	1836	0.757	93.92	0.85	54.02	94.09	98.19	292714806	26289175961
P11	10	6.76	405.6	399.9892	723	99.02%	98.85%	0.10%	13.7	246.6	0.807	90.57	1.71	54.69	95.03	98.56	222102864	19934568180
P12	20	23	1380	799.94	702	99.15%	98.36%	0.49%	76.8	1382.4	0.49	87.21	27.73	53.31	93.57	97.47	383408176	34200336392
P13	60	85.8	5148	799.656	703	97.31%	95.25%	0.07%	122	2196	0.65	93.59	0.15	48.43	95.45	98.64	395171786	35458890622
P14	50	50.6	3036	799.986	680	98.85%	98.69%	0.10%	40	720	0.869	93.84	1.82	61.25	93.91	98.08	273430268	24534462564
P15	80	75.6	4536	399.924	790	99.24%	98.12%	0.11%	118	2124	0.5	93.45	1.53	63.79	95.52	98.67	381180416	34181467933
P16	15	5.7	570	501.6	1024	97.85%	78.65%	0.19%	53.8	968.4	0.64	96.2	0.53	51.43	94.92	98.57	347743826	31235658201
P17	70	31.4	1884	800	531	98.21%	97.34%	0.06%	6.86	123.48	0.779	93.87	1.24	67.55	93.84	98.15	231598436	20791039839
P18	15	3.84	230.4	230.4	766	99.69%	98.69%	0.38%	45.2	813.6	0.862	93.75	1.44	59.29	95.04	98.63	408076212	36621793679
P19	10	72.23	7222.6	400.1542	782	99.82%	98.32%	0.15%	41.8	752.4	0.669	94.34	3.91	57.46	95.02	98.55	303220612	27230635854
P20	30	5.06	303.6	303.6	678	99.54%	98.84%	0.12%	16.1	289.8	0.851	95.4	1.21	49.55	94.11	98.27	284177748	25527342058
P21	10	68.8	8256	399.728	675	99.16%	98.79%	0.06%										
P22	20	10.9	654	400.03	592	99.49%	99.00%	0.20%	10.5	189	0.486	87.73	1.6	46	93.83	98.1	281281342	25251497145
P23	50	19.91	1991.3	799.9838	471	96.61%	87.68%	0.18%	108	1944	0.59	93.34	33.88	61.92	93	97.63	845650264	75855566553
P24	10	5.72	343.2	343.2	561	98.62%	97.98%	0.16%	16.7	300.6	0.43	84.9	2.59	47.24	94.61	98.3	446271092	39986686856
P25	20	52.4	3144	800.148	657	98.57%	98.48%	0.16%	54.6	982.8	0.773	93.67	1	51.71	93.7	98.06	427254012	38363299637

Supplementary Figure 1. Protein-protein interaction network of 709 differentially expressed genes between chemotherapy responders and non-responder. The label size and color of the nodes were determined according to their degree of interaction. The large label size and the red color represented higher degree.

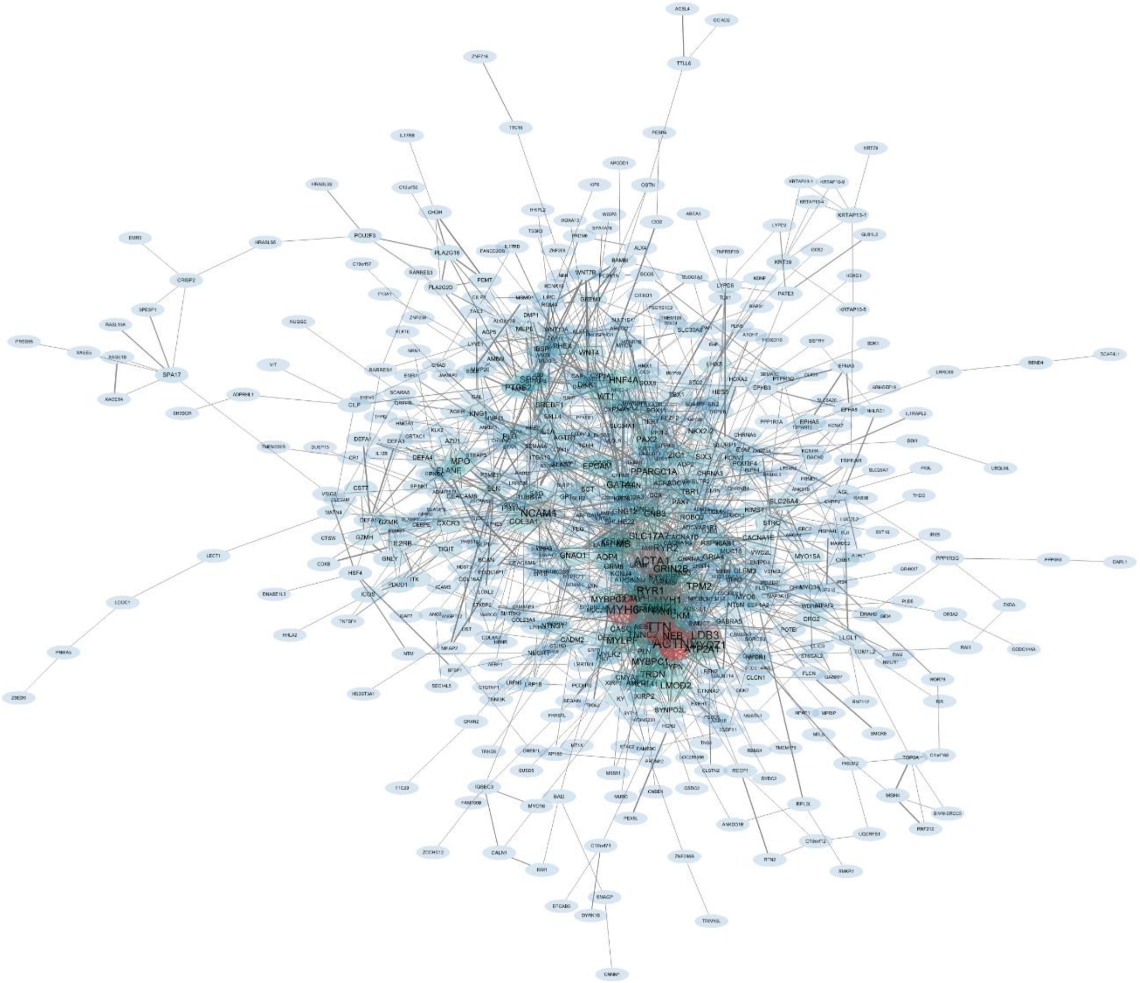

Supplement: Supplementary file 1 — Supplementary Information. [file 41598_2023_46857_MOESM1_ESM.pdf]
